# Long-Term Analysis of Resilience of the Oral Microbiome in Allogeneic Stem Cell Transplant Recipients

**DOI:** 10.3390/microorganisms10040734

**Published:** 2022-03-29

**Authors:** Alexa M. G. A. Laheij, Frederik R. Rozema, Michael T. Brennan, Inger von Bültzingslöwen, Stephanie J. M. van Leeuwen, Carin Potting, Marie-Charlotte D. N. J. M. Huysmans, Mette D. Hazenberg, Bernd W. Brandt, Egija Zaura, Mark J. Buijs, Johannes J. de Soet, Nicole N. M. Blijlevens, Judith E. Raber-Durlacher

**Affiliations:** 1 Department of Oral Medicine, Academic Centre for Dentistry Amsterdam, University of Amsterdam and Vrije Universiteit Amsterdam, 1081 LA Amsterdam, The Netherlands; fred.rozema@acta.nl (F.R.R.); j.raber.durlacher@acta.nl (J.E.R.-D.); 2 Department of Preventive Dentistry, Academic Centre for Dentistry Amsterdam, University of Amsterdam and Vrije Universiteit Amsterdam, 1081 LA Amsterdam, The Netherlands; b.brandt@acta.nl (B.W.B.); e.zaura@acta.nl (E.Z.); m.buijs@acta.nl (M.J.B.); j.d.soet@acta.nl (J.J.d.S.); 3 Department of Oral and Maxillofacial Surgery, Amsterdam UMC, University of Amsterdam, 1081 LA Amsterdam, The Netherlands; 4 Department of Oral Medicine, Atrium Health Carolinas Medical Centre, Charlotte, NC 28209, USA; mike.brennan@atriumhealth.org; 5 Department of Otolaryngology/Head and Neck Surgery, Wake Forest University School of Medicine, Winston-Salem, NC 27101, USA; 6 Department of Oral Microbiology and Immunology, Institute of Odontology, The Sahlgrenska Academy, University of Gothenburg, 413 90 Gothenburg, Sweden; ingervonb@hotmail.com; 7 Department of Dentistry, Radboud Institute for Health Sciences, Radboud University Medical Center, 6525 EX Nijmegen, The Netherlands; stephanie.vanleeuwen@radboudumc.nl (S.J.M.v.L.); marie-charlotte.huysmans@radboudumc.nl (M.-C.D.N.J.M.H.); 8 Department of Hematology, Radboud Institute for Health Sciences, Radboud University Medical Center, 6525 GA Nijmegen, The Netherlands; c.potting@hotmail.com (C.P.); nicole.blijlevens@radboudumc.nl (N.N.M.B.); 9 Department of Hematology, Amsterdam UMC, University of Amsterdam, 1081 LA Amsterdam, The Netherlands; m.d.hazenberg@amsterdamumc.nl

**Keywords:** allogeneic stem cell transplant, oral microbiome, dysbiosis, oral graft-versus-host disease, oral mucositis, conditioning

## Abstract

Stem cell transplantation (SCT) is associated with oral microbial dysbiosis. However, long-term longitudinal data are lacking. Therefore, this study aimed to longitudinally assess the oral microbiome in SCT patients and to determine if changes are associated with oral mucositis and oral chronic graft-versus-host disease. Fifty allogeneic SCT recipients treated in two Dutch university hospitals were prospectively followed, starting at pre-SCT, weekly during hospitalization, and at 3, 6, 12, and 18 months after SCT. Oral rinsing samples were taken, and oral mucositis (WHO score) and oral chronic graft-versus-host disease (NIH score) were assessed. The oral microbiome diversity (Shannon index) and composition significantly changed after SCT and returned to pre-treatment levels from 3 months after SCT. Oral mucositis was associated with a more pronounced decrease in microbial diversity and with several disease-associated genera, such as Mycobacterium, Staphylococcus, and Enterococcus. On the other hand, microbiome diversity and composition were not associated with oral chronic graft-versus-host disease. To conclude, dysbiosis of the oral microbiome occurred directly after SCT but recovered after 3 months. Diversity and composition were related to oral mucositis but not to oral chronic graft-versus-host disease.

## 1. Introduction

The oral cavity harbors a complex ecosystem of bacteria, fungi, viruses, archea, and protozoa. About 1000 different microbial species can be found in the oral cavity [1]. As bacteria in the oral cavity are mainly studied, much less is known about the other oral microorganisms [2]. The genus *Streptococcus* is most abundant in the oral cavity, while different niches additionally contain other genera, such as *Veillonella*, *Prevotella*, and *Fusobacterium*, depending on the substrate (for instance enamel, (un)keratinized mucosa, or saliva) and site in the oral cavity (for instance supra- or subgingival) [3]. The vast majority of the oral microorganisms live in biofilms [4].

The oral cavity becomes colonized rapidly after birth, while the oral microbiome establishes itself in close collaboration with the host’s immune system [2]. Oral mucosal cells are tolerant towards commensal microorganisms, while mucosal dendritic cells can mount an immune response against pathogenic microorganisms [5]. Other important factors that contribute to maintaining oral homeostasis include the chemical sensing of pathogenic microorganisms, salivary derived immunoglobulins (S-IgA), (glyco)proteins, and colonization resistance [2]. The oral microbiome is chemically and physically challenged several times a day, but despite these challenges the composition of the oral microbiome is fairly stable over time [6,7].

The role of the (oral) microbiome in health and disease has gained a lot of attention in recent years. It became clear that the microbiome is important for maintaining health. Our group and others studied the association between the oral microbiome and oral mucositis in autologous and allogeneic stem cell transplant recipients and acute graft-versus-host disease in allogeneic stem cell transplant patients.

In allogeneic stem cell transplantation, patients with hematological malignancies such as leukemia and lymphoma receive stem cells from a donor to induce a curative graft-versus-tumor effect; the immune cells of the donor will recognize the malignant cells as foreign and will destroy them. Allogeneic stem cell transplantation can lead to a vast array of side effects in the (peri)oral region, due to conditioning chemo- and/or radiotherapy or accompanying medication. These side effects include oral mucositis, hyposalivation, xerostomia, taste changes, oral infections and, when the donor immune system turns against the non-malignant cells of the recipient, oral graft-versus-host disease. They may lead to oral pain, hypersensitivity of the oral mucosa, and difficulty in eating, drinking, and performing proper oral hygiene, which in turn may lead to progression of dental caries and poor quality of life [8].

A number of studies described changes in the oral microbiome early after cancer treatment [9,10,11,12,13,14,15]. Several genera, including *Staphylococcus* and *Enterococcus*, and loss of microbiome diversity were associated with oral mucositis [9,10,12,16]. However, the prospective long-term data linking of the oral microbiome to oral mucositis and oral chronic graft-versus-host disease in stem cell transplantation recipients is still very scarce. Therefore, the aim of our study was to longitudinally study the oral microbiome in allogeneic stem cell transplant recipients and to relate the oral microbiome to oral mucositis and oral chronic graft-versus-host disease.

## 2. Materials and Methods

This study was performed as an ancillary study of the Orastem study, an international, observational, prospective study into the impact of oral complications after stem cell transplantation [17]. This study was approved by the Medical Research Ethical Committee from Amsterdam University Medical Centers location AMC and the Radboud University Medical Center Nijmegen (NL52117.018.15) and is registered in the Netherlands Trial Register (NL5645). The study was funded by the Dutch Cancer Foundation (ACTA 2014-7468). The study was carried out in accordance with the relevant guidelines and regulations. All patients provided written informed consent. The data on the oral microbiome, salivary proteome, and oral mucositis in autologous SCT patients from the same study are reported elsewhere [10,18]. The demographic and other relevant data were retrieved from the medical charts.

### 2.1. Study Population

Adult patients that had received an allogeneic stem cell transplant (SCT) in Amsterdam UMC, location AMC and Radboud UMC, the Netherlands, between September 2015 and June 2018 were eligible for inclusion into this study. Patients were excluded if they were edentulous. Before SCT, all the participants received a full dental examination. In patients that consented, evident oral foci (e.g., semi-impacted third molars, deep caries lesions, periapical lesions, and deep periodontal pockets) were eliminated as much as possible before SCT. All patients entered SCT without oral symptoms. During hospitalization, institutional oral care protocols (not including chlorhexidine rinses) were followed. Patients received antiviral and anti-fungal prophylaxis. Standard antibiotic prophylaxis regimens consisted of ciprofloxacin and cotrimoxazole. In addition, patients received GvHD prophylaxis according to local protocols.

### 2.2. Sample Collection and Clinical Measurements

To collect oral samples, participants were asked to rinse the oral cavity thoroughly for 20–30 s with 10 mL of sterile 0.9% saline solution. The solution was collected in a sterile tube, kept on ice, and centrifuged at 4500× *g* for 7 min within two hours. Pellets were resuspended in 1 mL sterile PBS and stored at −80 °C until analysis.

After SCT, during neutropenia, oral mucositis was scored according to the criteria of the WHO by calibrated researchers [19]. Scores were recalculated into no ulcerative oral mucositis (WHO 0–1) and ulcerative oral mucositis (WHO 2–4). After SCT, oral chronic graft-versus-host disease (GVHD) was scored according to the criteria of the NIH [20]. The severity of three oral manifestations (erythema, lichenoid changes, and ulcerations) was scored by trained dentists. Total oral chronic GVHD score ranged from 0 to 12, and for this study, a score of 2 and higher was categorized as oral chronic GVHD. Clinical assessment and oral rinsing sampling took place from 8 weeks to days before SCT, weekly during hospitalization (between 2 and 4 weeks after SCT), and 3, 6, 12 and 18 months after SCT. Oral rinsing samples were collected three times a week, but for this analysis one sample per week was chosen, i.e., from the same day as the saliva samples were collected (as part of another component of this study).

### 2.3. Sequencing of Oral Rinsing Samples

The oral rinsing samples were thawed and pelleted by centrifugation. The pellets were resuspended in 100 µL TE buffer (Tris-EDTA) and transferred to a 96-well deepwell plate. After addition of 100 µL Lysis buffer (LGC genomics GmbH, Berlin, Germany), 250 µL 0.1mm Zirconia beads (Biospec, Bartlesville, OK, USA), and 200 µL RotiPhenol (Carl Roth, Karlsruhe, Germany), the mixture was subjected to four bead beating steps of 2 min each. DNA extraction and purification was performed with the LGC Magmini kit (LGC genomics GmbH), after which the bacterial DNA concentration was determined by a 16S ribosomal DNA gene quantitative polymerase chain reaction (qPCR) [21]. For each sample, 2 ng of DNA was amplified, with barcoded forward and reverse primers [22], using the 16S rRNA gene-specific sequences V4F/515F: GTGCCAGCMGCCGCGGTAA and V4R/806R: GGACTACHVGGGTWTCTAAT [23]. From the final amplicon mix, 8 pmol, including 30% PhiX, was added. This V4 hypervariable region of the 16S rRNA gene was sequenced using the Illumina MiSeq platform with the MiSeq Reagent Kit v3 (251 nucleotides paired end) at the Core Facility Genomics, Amsterdam UMC.

The reads were quality-filtered, denoised, mapped to zero-radius operational taxonomic units (zOTUs), and assigned taxonomy using the HOMD database (v14.51) [24], as described earlier [25]. Control samples consisted of PBS solution, sterile 0.9% NaCl solution, blank DNA isolations, and PCR controls. All 29 control samples had extremely low read output (median: 8 reads/sample, Table S1). As no contaminants were identified, the control samples did not need to be considered further. The final zOTU table was randomly subsampled at 9350 reads/sample. No patients’ samples were lost because of subsampling.

### 2.4. Statistical Analysis

Principal component analysis (PCA), permutational multivariate analysis of variance (PERMANOVA) using the Bray-Curtis similarity distance, and 9999 permutations, was calculated using PAST version 4.08 [26], after log-2 transformation of the subsampled zOTU table. PERMANOVA with permutations restricted within the subject (for dependent) samples was performed using R v4.1.2 [27] and the packages vegan v2.5–7 [28], microbiome v1.14.0 [29], and phyloseq v1.36.0 [30]. The Shannon diversity index was calculated using the diversity function in the microbiome R-package. A *p*-value < 0.001 was considered statistically significant.

The relations between independent variables and the longitudinally measured diversity (Shannon index) of the oral microbiome were analyzed using Linear Mixed Model Analysis for continuous outcome values, to correct for the dependency in outcome measurement. Independent variables were entered as fixed effects. The relation between the presence of oral mucositis and oral GVHD was calculated using the Chi-square test. SPSS version 26 (IBM) was used for Chi-square and LMM analyses. A *p*-value < 0.05 was considered statistically significant.

## 3. Results

### 3.1. Patient Characteristics

For the Orastem study, 67 Dutch allogeneic SCT patients were included. For this study, the allogeneic SCT patients were selected from those from whom an oral rinsing sample was available pre-SCT, and from whom, at least two samples were available from three months after SCT. That resulted in 50 patients: 10 from Amsterdam UMC and 40 from Radboud UMC; 52% were female, and 48% were male. Their age ranged from 19 to 74 with an average of 52.0 years. Most patients were diagnosed with acute myeloid leukemia and had received a conditioning regimen before allogeneic SCT, consisting of cyclosporin as a GVHD prophylaxis. The conditioning regimens of 20 patients were classified as non-myeloablative, 18 as reduced intensity, and 12 as myeloablative. The source of the donor cells was most often a matched unrelated donor (68%) (Table 1). One patient smoked, while 29 had quit smoking, and 20 were never-smokers. Of all the patients, 10 (20%) developed ulcerative oral mucositis, whereas in 15 (30%) patients, oral chronic graft-versus-host disease manifested during the study.

### 3.2. Microbiome Analyses

There were on average 17286.6 (±3984.8) reads per sample. After subsampling, the 1085 zOTUs were classified into 123 different genera or higher taxa. The top five most abundant zOTUs were classified as: Streptococcus dentisani*, *Veillonella*, *Streptococcus salivarius/vestibularis*, *Prevotella*, and *Neisseria flavescens/subflava*. In total, there were 316 samples available for microbial analysis after subsampling. Per sampling time in the study, these number of samples were available: 50 pre-SCT, 42 in week 1 after SCT, 33 in week 2 after SCT, 22 in week 3 after SCT, 37 three months after SCT, 47 six months after SCT, 43 samples 12 months after SCT, and 42 samples 18 months after SCT. Samples were missing because patients were discharged from the hospital (in the weeks after SCT), patients missed an appointment for the study, or patients died.

At the baseline, there was no difference in microbial composition or alpha-diversity based on conditioning regimens, hospital, gender, and smoking habits and between individuals who did and did not develop ulcerative oral mucositis and those who did and did not develop oral chronic graft-versus-host disease (PERMANOVA, *p* > 0.001; Mann–Whitney U test, *p* > 0.05).

**Figure 1:**
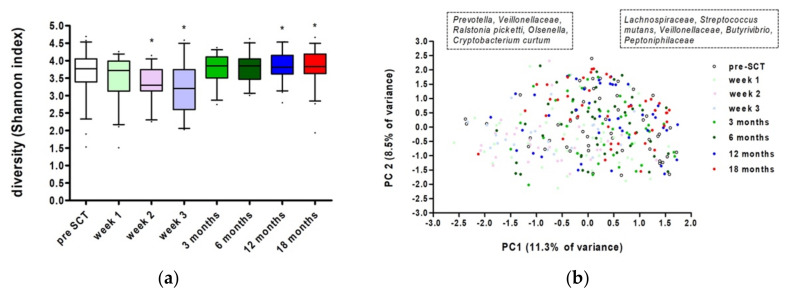
Oral microbiome at all time points in the study: (**a**) Shannon diversity index (boxes show median and whiskers 5–95 percentile). * Marks significant differences compared to pre-SCT (Linear Mixed Model Analysis, *p* < 0.05); (**b**) principal component analysis plot. Left box indicates more abundant taxa at low values of the loadings of PC1, right box—at high values.

There was a significant effect of time on bacterial diversity for the whole group (Linear Mixed Model, *p* = 0.0000013096). Compared to the Shannon diversity index at pre-SCT, there was a significantly lower diversity at week 1 (*p* = 0.043), week 2 (*p* = 0.002), and week 3 (*p* = 0.000085). At three months, the microbial diversity had reached pre-treatment levels. Moreover, there was a slight, but significant increase in diversity at 12 (*p* = 0.024) and 18 months (*p* = 0.029) after SCT (Figure 1a). Bacterial diversity could not be explained by treatment center, gender, age, (un)stimulated salivary flow, neutrophil or leukocyte count, myelotoxicity of the conditioning regimen, or the donor source (Linear Mixed Model Analysis, *p* > 0.05).

Besides an effect on diversity, there was also a significant effect of time on the oral microbial composition of the whole group at the zOTU level (restricted PERMANOVA, F = 2.511, *p* = 0.00001, Figure 1b). The composition changed, directly after allogeneic SCT, remained different for at least two to three weeks, returned to pre-treatment conditions after three months, and remained stable until 18 months after SCT. Samples from pre-SCT and 3, 6, 12, and 18 months after SCT had a higher relative abundance of *Lachnospiraceae*, *Streptococcus mutans*, *Veillonellaceae*, *Butyrivibrio*, and *Peptoniphilaceae*. The oral microbiome in the first weeks after SCT showed a higher relative abundance of *Prevotella*, *Veillonellaceae*, *Ralstonia picketti*, *Olsenella*, and *Cryptobacterium curtum*.

### 3.3. Ulcerative Oral Mucositis

**Figure 2:**
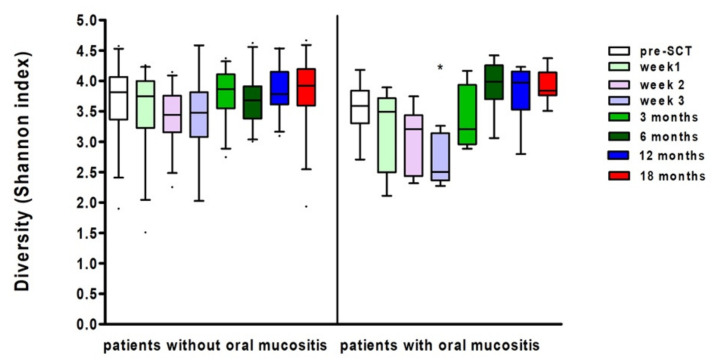
Shannon diversity index at several time points in patients with and without oral mucositis (boxes show median and whiskers 5-95 percentile). * Marks significant differences compared to the other patient group (Linear Mixed Model Analysis, *p* < 0.05).

Ten patients developed ulcerative oral mucositis (WHO grade > 2), in ten patients the WHO score was missing. In eight patients, the oral mucositis score was missing. There was a significant effect of oral mucositis on bacterial diversity (Linear Mixed Model Analysis, *p* = 0.028). Bacterial diversity was lower when oral mucositis was present. While microbial diversity on average decreased after SCT, the effect over time was different for patients who did and did not develop oral mucositis. (Linear Mixed Models, *p* = 0.013, see Figure 2). At three weeks after SCT, the decrease in microbial diversity was significantly larger for patients who did develop oral mucositis compared to patients who did not develop oral mucositis (Linear Mixed Models, *p* = 0.032). The standard deviation in the patients with oral mucositis was at some time points considerable. When the neutrophil count was added in the regression model, it turned out that the neutrophil count was a confounding variable, influencing both oral mucositis and microbial diversity (Linear Mixed Model Analysis, *p* = 0.048), while the neutrophil count had no direct effect on diversity (Linear Mixed Model Analysis, *p* = 0.098).

**Figure 3:**
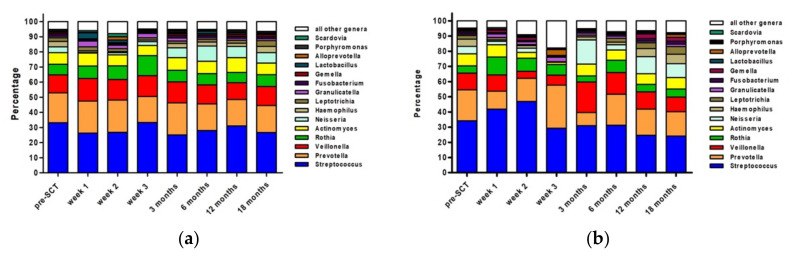
Relative Abundance (percentage) of top 15 most abundant genera at different time points in patients who did not experience oral mucositis (**a**) and patients who did experience oral mucositis (**b**).

The relative abundance of the top 15 genera over all time points in the study is shown in Figure 3. In general, the relative abundance of the dominant genera remained fairly stable in patients who did not experience oral mucositis (Figure 3a). The most prominent effect was the increase in *Rothia* at the expense of *Prevotella* at week 3. Moreover, *Neisseria* decreased after SCT, recovered at 3 weeks, and increased thereafter.

In patients who did develop oral mucositis, the relative abundance of the top 15 genera was less stable (Figure 3b). Most obvious changes could be seen at three weeks and three months after SCT. At three weeks after SCT, *Streptococcus* was lower in abundancy and *Neisseria* was almost absent, while *Prevotella* and *Alloprevotella* were more abundant. Most striking was the increase in the ‘other’ group. This group consisted almost solely of *Mycobacterium* (5.1%), *Staphylococcus* (4.8%), and *Enterococcus* (3.4%). While *Staphylococcus* was also present at week two after SCT in patients with oral mucositis (3.5%), at the other time points this genus was almost absent, as was the case for all other time points for *Mycobacterium* and *Enterococcus*. At three months after SCT, the high relative abundance of *Neisseria* and *Veillonella* at the expense of *Prevotella* and the top 7–15 genera stands out. On an individual level, there were no patients with a domination (>30% of relative abundance) of *Mycobacterium*, *Staphylococcus*, or *Enterococcus* in the weeks after SCT.

**Figure 4:**
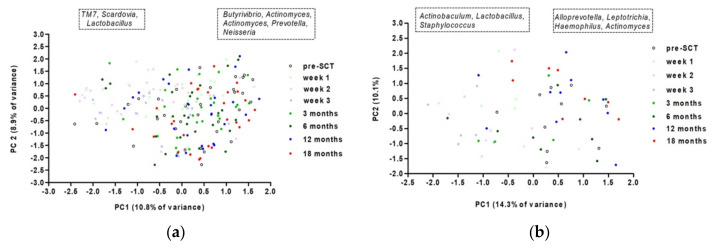
PCA on oral microbiome over time for patients who did not (**a**) and did (**b**) experience oral mucositis; left box more abundant taxa at low values of PC1 loadings; right box more abundant taxa at high values of PC1.

In patients with and without OM there was a significant effect of time on the oral microbial composition on the zOTU level (restricted PERMANOVA, F = 1.7926, *p* = 0.0001, restricted PERMANOVA, F = 1.5376, *p* = 0.0001, Figure 4). However, the zOTUs with the highest (absolute) loadings on PC1 are different between both groups. After SCT, TM7 (*Saccharibacteria*), *Scardovia*, and *Lactobacillus* were more abundant in patients without oral mucositis, while *Actinobaculum*, *Lactobacillus*, and *Staphylococcus* were more abundant in patients with oral mucositis (Figure 4).

### 3.4. Oral Chronic Graft-versus-Host Disease

**Figure 5:**
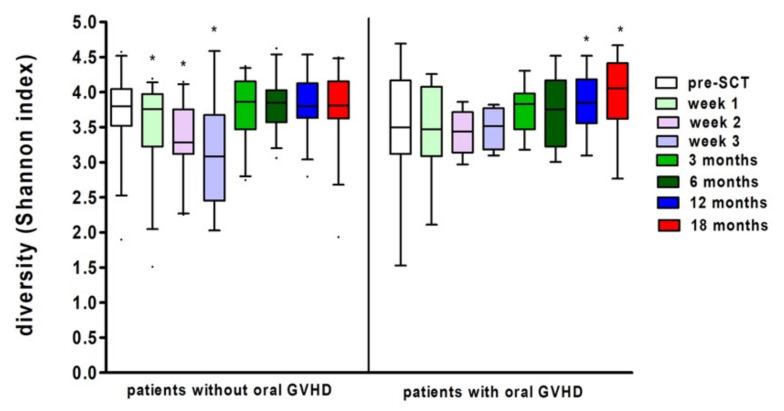
Shannon diversity index at several time points in patients with and without oral GVHD (median, 5–95 percentile). * Marks significant differences compared to pre-SCT (Linear Mixed Model Analysis, *p* < 0.05).

In our study, 15 patients (30%) developed oral chronic GVHD at any time after SCT. There was no significant association between patients who did/did not develop oral GVHD at any time and microbial diversity (Linear Mixed Model Analysis, *p* = 0.451). Moreover, the development of the bacterial diversity over time was not significantly different for (non-) oral chronic graft-versus-host disease patients (Linear Mixed Model Analysis, *p* = 0.497). Yet, in both groups there was a significant effect of time on microbial diversity, although the trend differed (Figure 5). In the patients without oral chronic graft-versus-host disease, there was a significant decrease in diversity at weeks 1, 2, and 3 (Linear Mixed Model Analysis, *p* = 0.037; *p* = 0.002; *p* < 0.001). While in patients with oral chronic graft-versus-host disease, there was a slight, yet significant, increase in diversity at 12 and 18 months (Linear Mixed Model Analysis, *p* = 0.048; *p* = 0.017).

**Figure 6:**
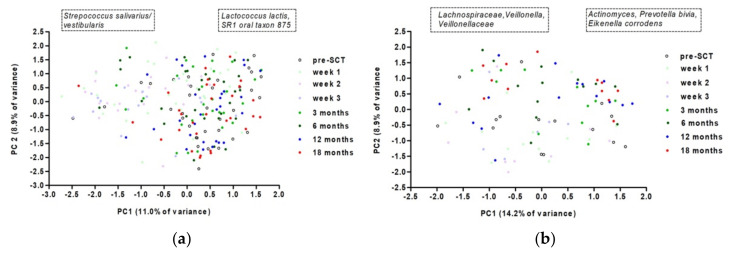
Principal component analysis on oral microbiome over time for patients who did not (**a**) and did (**b**) experience oral GVHD. Left box—more abundant taxa at low values of PC1 loadings, right box—at high values.

In patients who did and did not develop oral chronic graft-versus-host disease during the duration of the study, there was a significant effect of time on microbiome composition (restricted PERMANOVA, F = 1.1191, *p* = 0.0001; F = 2.1962, *p* = 0.0001, Figure 6). The change in microbiome composition did not coincide with the time that oral chronic graft-versus-host disease was present, as the changes in microbiome were visible after SCT, while oral chronic graft-versus-host disease appeared from three months after SCT.

In patients who developed oral chronic graft-versus-host disease after SCT, there was no difference in microbial composition between pre-SCT samples and the samples taken at the time they developed oral chronic graft-versus-host disease (restricted PERMANOVA, F = 1.0316, *p* = 0.0341).

There was no significant association between the presence of ulcerative oral mucositis and oral GVHD (Chi-square, *p* = 0.410), meaning that in our study patients who experienced oral mucositis did not have a higher risk of having oral GVHD and vice versa (Table 2).

## 4. Discussion

The aim of our study was to longitudinally assess the oral microbiome in allogeneic stem cell transplant recipients and to relate the oral microbiome to oral mucositis and oral chronic graft-versus-host disease. The diversity and composition of the oral microbiome significantly changed after SCT, returned to pre-treatment levels from three months after SCT, and remained fairly stable until 18 months after SCT. Bacterial diversity was lower when oral mucositis was present, while there was no significant relationship between diversity and the presence or absence of oral chronic graft-versus-host disease. Changes in the microbiome composition after SCT were different for patients who did and did not experience oral mucositis, while changes in the microbiome composition did not coincide with the presence of oral chronic graft-versus-host disease.

The decrease in oral bacterial diversity after SCT has been described by our group after autologous SCT [10] and by others in both adults and children in alloSCT and autoSCT patients [9,14,15,16,31,32,33]. Most studies described a significant change in microbial composition directly after SCT. Changes in the oral microbiome were related to oral mucositis in adults [9,10,16,31,32] or to acute GVHD anywhere in the body in adult patients [14] or children [15]. Almost all the studies lack data on the recovery of the oral microbiome as these studies ended at three to four weeks after SCT. At that time, the oral microbiome is most different from pre-SCT conditions. The present study, as well as our previous study in autoSCT recipients and the study of Ingham et al., showed that the oral microbiome is resilient as it returns to pre-SCT levels three months after SCT, remaining relatively stable in the first 12 to 18 months following SCT [10,15].

In the first weeks after SCT, when the defense mechanisms of the host were seriously hampered because of neutropenia and conditioning therapy, all the characteristics of microbial dysbiosis [34] were present: the overall loss of microbial diversity; the loss of commensal microbes (*Streptococcus* and *Veillonella*); and the expansion of pathogenic microbes (*Mycobacterium*, *Staphylococcus*, and *Enterococcus*). *Staphylococcus* and *Enterococcus* are Gram-positive bacteria that possess several virulence factors allowing them to cause infections at many different body sites. Other studies found an association of these genera with oral mucositis as well, either as an increase in relative abundance, or as discriminating taxa after SCT in patients with oral mucositis [9,10,16,32].

Some less-known taxa were more abundant in samples taken in the first weeks after SCT, compared to pre-SCT samples and samples taken at 3, 6, 12, and 18 months after SCT. Bacteria present in the oral cavity during the first weeks after SCT included *Ralstonia pickettii*, which is a Gram-negative, aerobic bacterium of the genus *Pseudomonas.* This microorganism is mostly present in the soil, but may be transmitted via fluids and contaminated medical products [35]. It is considered a low pathogenic bacterium, but it is described to be a causative agent of systemic infections in hospitalized patients. Moreover, *Ralstonia pickettii* was associated with lower survival after SCT [14,36]. *Olsenella* is a Gram-positive, rod-shaped genus, first isolated from the oral cavity. It has been found in endodontic infections [37] and in active caries lesions [38,39]. *Cryptobacterium curtum* is a Gram-positive, obligately anaerobic, rod-shaped bacterium that has been associated with dental abscesses and periodontitis [40,41].

The change in microbial composition in oral mucositis patients was characterized by a higher abundance of disease-associated bacteria such as *Actinobaculum* (also known as *Actinotignum*) [42], *Lactobacillus*, and *Staphylococcus*. The microbial changes in patients that did not experience oral mucositis were characterized by caries-associated bacteria TM7 (*Saccharibacteria)*, *Scardovia*, and *Lactobacillus*, as the Gram positive *Scardovia* and *Lactobacillus* are acid-producing bacteria [43]. *Saccharibacteria* are parasitic bacteria, probably modulating the oral microbiome structure hierarchy and functionality [44].

We found no association between the diversity of the oral microbiome and the presence or absence of oral chronic graft-versus-host disease. Moreover, there was no difference in microbial composition between the oral samples taken pre-SCT and at the time when oral chronic graft-versus-host disease was present. Changes in microbiome composition did not coincide with oral chronic graft-versus-host disease. Heidrich et al. also did not find an association between acute GVHD anywhere in the body and the microbiome [14]. However, they described a higher acute GVHD risk in patients with a domination of *E. faecalis*. Ingham et al. also found oral bacteria as predictors for acute GVHD anywhere in the body in children; however, they reported different taxa to Heidrich et al., namely *Actinomyces* spp and *Prevotella melaninogenica* [15]. In our study, none of the patients showed a domination of any single taxa (data not shown). Campos de Molla et al. studied the relationship between oral microbial diversity and oral chronic graft-versus-host disease, and they did not find a relationship either [32].

As graft-versus-host disease has an inflammatory component, it was hypothesized that a dysbiotic state of the oral microbiome might predispose to the development of oral chronic graft-versus-host disease. As immunosuppressive and antibiotic medication might have influenced either the oral microbiome or the graft-versus-host disease, we looked for differences in the use of these medications between patients who did and did not have oral chronic graft-versus-host disease. However, there was no pattern detectable in medication use in relation to the presence and absence of oral chronic graft-versus-host disease (data not shown). It could be that the heterogenicity of the alloSCT patient group and the relative low number of patients with oral chronic graft-versus-host disease (*n* = 15) may have underpowered our study. In that case, a study with a larger number of patients is needed. However, in our study only 10 patients experienced oral mucositis, and an association with the oral microbiome was assessed, suggesting more heterogeneous microbiome changes in oral chronic graft-versus-host disease cases than in cases with oral mucositis. On the other hand, it could also be that the development of oral chronic graft-versus-host disease is more strongly driven by other factors, such as the source of the stem cells, age, prior acute GVHD, genomics, and the match between donor and recipient, dominating the oral microbiome.

At the end of the study, there was a slight, but significant, increase in the diversity of the oral microbiome, compared to pre-SCT. In the results of Ingham et al. [15], this pattern was also found, although they did not report or test the significance of this finding. It is not clear whether this was a true increase or whether it was an incidental finding. In the case of a true finding, it could be that the pre-SCT diversity was lower than the average diversity in a healthy population, or, that the diversity in our patients was lower before SCT, compared to before they got ill. In that case, the oral microbiome is influenced not only by the SCT, but also by the disease or the previous treatments for the disease. If the disease gets under control, it could be that the oral microbiome gets healthier as well. To test this hypothesis, data covering years after SCT should be gathered, combined with data on the disease state and oral health, as data on the state of the oral microbiome before patients get ill will not be available. Another option would be to compare the data with a healthy age- and gender-matched population.

In this, and our previous study in SCT patients [10], oral rinsing samples were collected to determine the oral microbiome. In order to link the microbiome to signs of the oral mucosa, a sampling method representing the whole oral cavity would suit best, and saliva would be the method of choice. However, many SCT patients experience (severe) hyposalivation at a certain timepoint, making it impossible to collect enough saliva at every timepoint to determine reliable and accurate microbiome profiles. As rinsing the oral cavity with a sterile and neutral solution leads to microbial profiles similar to those in saliva samples [45], this method was chosen.

A limitation of our study is the heterogenicity of the patient group. Patients were (more or less) heterogeneous in diagnosis, conditioning regimen, donor source, antimicrobials and other medication used, and age. Although no differences between the various groups were seen at the baseline, each of these factors might have had an influence on the outcome of our study throughout the duration of the study. Moreover, interaction effects were a possibility. At some timepoints, the standard deviation was quite considerable, while the reason for this spreading was not clear. Larger study groups in more homogenous groups would be preferable, making it possible to construct subgroups of patients with similar diagnosis, treatment, age, and antimicrobial use.

## 5. Conclusions

The oral microbiome changed significantly in the weeks after SCT, leading to a state of dysbiosis; yet, it showed recovery as a microbiome composition, and diversity returned to pre-SCT levels after three months and remained stable until 18 months after SCT. The diversity and composition of the oral microbiome were related to oral mucositis, but no clear link with oral chronic graft-versus-host disease was found.

## Figures and Tables

**Table 1:** Patient characteristics (*n* = 50).

Conditioning Regimens		Diagnoses		Donor Source		GVHD Prophylaxis	
ATG, Bu, Flu	11	Acute myeloid leukemia	19	Matched unrelated donor	34	Cyclosporin	33
Flu, TBI	10	Myelodysplastic syndrome	7	Sibling	10	Cyclosporine and methotrexate	6
ATG, Cy, TBI	7	Lymphoma	6	Mismatched unrelated donor	2	Cyclosporine and other	6
ATG, Flu, TBI	6	Acute lymphoid leukemia	4	Female donor	2	Cyclosporin and prednisolone	1
ATG, Flu, TBI	4	Chronic lymphoid leukemia	2	Unknown	2	Cyclosporine, methotrexate, and anti-thymocyte globulin	1
ATG, Cy	3	Severe aplastic anemia	2			Cyclosporin and T-cell depleted transplant	1
Cy, TBI	3	Myelofibrosis	2			Cyclosporine, methotrexate, and prednisolone	1
Flu, TBI, Cy	2	Myeloma	2			Tacrolimus and prednisolone	1
Other	4	Chronic lymphoid leukemia	1				
ATG, Bu, Flu	11	Other	5				

ATG = antithymocyte globuline, Bu = busulfan, Cy = cyclophosphamide, Flu = fludarabine, TBI = total body irradiation.

**Table 2:** Distribution of patients who did and did not develop oral mucositis vs patients who did and did not develop oral GVHD.

		Developed Oral Mucositis?		
		no	Yes	
**Developed oral GVHD?**	no	25	6	31
	yes	7	4	11
**Total**		32	10	42

Oral mucositis score was missing in 10 patients.

## Data Availability

The data presented in this study are available on request from the corresponding author. The data are not publicly available due to privacy issues regarding the metadata used.

## Supplementary Materials

The following are available online at https://www.mdpi.com/article/10.3390/microorganisms10040734/s1, Table S1: Sequencing output control samples.

## References

[1] Dewhirst F.E., Chen T., Izard J., Paster B.J., Tanner A.C., Yu W.H., Lakshmanan A., Wade W.G. (2010). The Human Oral Microbiome. J. Bacteriol..

[2] Zaura E., Nicu E.A., Krom B.P., Keijser B.J. (2014). Acquiring and Maintaining a Normal Oral Microbiome: Current Perspective. Front. Cell. Infect. Microbiol..

[3] Li K., Bihan M., Methé B.A. (2013). Analyses of the Stability and Core Taxonomic Memberships of the Human Microbiome. PLoS ONE.

[4] Berger D., Rakhamimova A., Pollack A., Loewy Z. (2018). Oral Biofilms: Development, Control, and Analysis. High Throughput.

[5] Novak N., Haberstok J., Bieber T., Allam J.P. (2008). The Immune Privilege of the Oral Mucosa. Trends Mol. Med..

[6] Tamashiro R., Strange L., Schnackenberg K., Santos J., Gadalla H., Zhao L., Li E.C., Hill E., Hill B., Sidhu G. (2021). Stability of Healthy Subgingival Microbiome across Space and Time. Sci. Rep..

[7] Kruse A.B., Schlueter N., Kortmann V.K., Frese C., Anderson A., Wittmer A., Hellwig E., Vach K., Al-Ahmad A. (2021). Long-Term Use of Oral Hygiene Products Containing Stannous and Fluoride Ions: Effect on Viable Salivary Bacteria. Antibiotics.

[8] Haverman T.M., Raber-Durlacher J.E., Rademacher W.M., Vokurka S., Epstein J.B., Huisman C., Hazenberg M.D., de Soet J.J., de Lange J., Rozema F.R. (2014). Oral Complications in Hematopoietic Stem Cell Recipients: The Role of Inflammation. Mediat. Inflamm..

[9] Shouval R., Eshel A., Dubovski B., Kuperman A.A., Danylesko I., Fein J.A., Fried S., Geva M., Kouniavski E., Neuman H. (2020). Patterns of Salivary Microbiota Injury and Oral Mucositis in Recipients of Allogeneic Hematopoietic Stem Cell Transplantation. Blood Adv..

[10] Laheij A.M.G.A., Raber-Durlacher J.E., Koppelmans R.G.A., Huysmans M.D.N.J.M., Potting C., van Leeuwen S.J.M., Hazenberg M.D., Brennan M.T., von Bültzingslöwen I., Johansson J.E. (2019). Microbial Changes in Relation to Oral Mucositis in Autologous Hematopoietic Stem Cell Transplantation Recipients. Sci. Rep..

[11] Laheij A.M., de Soet J.J., von dem Borne P.A., Kuijper E.J., Kraneveld E.A., van Loveren C., Raber-Durlacher J.E. (2012). Oral Bacteria and Yeasts in Relationship to Oral Ulcerations in Hematopoietic Stem Cell Transplant Recipients. Support. Care Cancer.

[12] Diaz P.I., Hong B.Y., Frias-Lopez J., Dupuy A.K., Angeloni M., Abusleme L., Terzi E., Ioannidou E., Strausbaugh L.D., Dongari-Bagtzoglou A. (2013). Transplantation-Associated Long-Term Immunosuppression Promotes Oral Colonization by Potentially Opportunistic Pathogens without Impacting Other Members of the Salivary Bacteriome. Clin. Vaccine Immunol..

[13] Hou J., Zheng H.M., Li P., Liu H.Y., Zhou H.W., Yang X.J. (2018). Distinct Shifts in the Oral Microbiota Are Associated with the Progression and Aggravation of Mucositis During Radiotherapy. Radiother. Oncol..

[14] Heidrich V., Bruno J.S., Knebel F.H., de Molla V.C., Miranda-Silva W., Asprino P.F., Tucunduva L., Rocha V., Novis Y., Arrais-Rodrigues C. (2021). Dental Biofilm Microbiota Dysbiosis Is Associated with the Risk of Acute Graft-Versus-Host Disease after Allogeneic Hematopoietic Stem Cell Transplantation. Front. Immunol..

[15] Ingham A.C., Kielsen K., Mordhorst H., Ifversen M., Aarestrup F.M., Müller K.G., Pamp S.J. (2021). Microbiota Long-Term Dynamics and Prediction of Acute Graft-Versus-Host Disease in Pediatric Allogeneic Stem Cell Transplantation. Microbiome.

[16] Lee A., Hong J., Shin D.Y., Koh Y., Yoon S.S., Kim P.J., Kim H.G., Kim I., Park H.K., Choi Y. (2020). Association of Hsv-1 and Reduced Oral Bacteriota Diversity with Chemotherapy-Induced Oral Mucositis in Patients Undergoing Autologous Hematopoietic Stem Cell Transplantation. J. Clin. Med..

[17] Brennan M.T., Hasseus B., Hovan A.J., Raber-Durlacher J.E., Blijlevens N.M., Huysmans M.C., Legert K.G., Johansson J.E., Moore C.G., von Bultzingslowen I. (2018). Impact of Oral Side Effects from Conditioning Therapy before Hematopoietic Stem Cell Transplantation: Protocol for a Multicenter Study. JMIR Res. Protoc..

[18] Van Leeuwen S.J.M., Proctor G.B., Laheij A.M.G.A., Potting C.M.J., Smits O., Bronkhorst E.M., Hazenberg M.D., Haverman T.M., Brennan M.T., von Bültzingslöwen I. (2021). Significant Salivary Changes in Relation to Oral Mucositis Following Autologous Hematopoietic Stem Cell Transplantation. Bone Marrow Transplant..

[19] World Health Organization (1979). Handbook for Reporting Results of Cancer Treatment.

[20] Elad S., Zeevi I., Or R., Resnick I.B., Dray L., Shapira M.Y. (2010). Validation of the National Institutes of Health (Nih) Scale for Oral Chronic Graft-Versus-Host Disease (Cgvhd). Biol. Blood Marrow Transplant..

[21] Ciric L., Pratten J., Wilson M., Spratt D. (2010). Development of a Novel Multi-Triplex Qpcr Method for the Assessment of Bacterial Community Structure in Oral Populations. Environ. Microbiol. Rep..

[22] Kozich J.J., Westcott S.L., Baxter N.T., Highlander S.K., Schloss P.D. (2013). Development of a Dual-Index Sequencing Strategy and Curation Pipeline for Analyzing Amplicon Sequence Data on the Miseq Illumina Sequencing Platform. Appl. Environ. Microbiol..

[23] Gregory C.J., Lauber C.L., Walters W.A., Berg-Lyons D., Lozupone C.A., Turnbaugh P.J., Fierer N., Knight R. (2011). Global Patterns of 16s Rrna Diversity at a Depth of Millions of Sequences Per Sample. Proc. Natl. Acad. Sci. USA.

[24] Chen T., Yu W.H., Izard J., Baranova O.V., Lakshmanan A., Dewhirst F.E. (2010). The Human Oral Microbiome Database: A Web Accessible Resource for Investigating Oral Microbe Taxonomic and Genomic Information. Database.

[25] Koopman J.E., Buijs M.J., Brandt B.W., Keijser B.J., Crielaard W., Zaura E. (2016). Nitrate and the Origin of Saliva Influence Composition and Short Chain Fatty Acid Production of Oral Microcosms. Microb. Ecol..

[26] Hammer Ø., Harper D.A.T., Ryan P.D. (2001). Past: Paleontological Statistics Software Package for Education and Data Analysis. Palaeontol. Electron..

[27] (2015). R: A Language and Environment for Statistical Computing.

[28] (2020). Vegan: Community Ecology Package.

[29] Lahti L., Shetty S. (2017). Tools for Microbiome Analysis in R. http://microbiome.github.com/microbiome.

[30] McMurdie P.J., Holmes S. (2013). Phyloseq: An R Package for Reproducible Interactive Analysis and Graphics of Microbiome Census Data. PLoS ONE.

[31] Takahashi M., Toyosaki M., Matsui K., Machida S., Kikkawa E., Ota Y., Kaneko A., Ogawa Y., Ando K., Onizuka M. (2020). An Analysis of Oral Microbial Flora by T-Rflp in Patients Undergoing Hematopoietic Stem Cell Transplantation. Int. J. Hematol..

[32] De Molla V.C., Heidrich V., Bruno J.S., Knebel F.H., Miranda-Silva W., Asprino P.F., Tucunduva L., Rocha V., Novis Y., Camargo A.A. (2021). Disruption of the Oral Microbiota Is Associated with a Higher Risk of Relapse after Allogeneic Hematopoietic Stem Cell Transplantation. Sci. Rep..

[33] Badia P., Andersen H., Haslam D., Nelson A.S., Pate A.R., Golkari S., Teusink-Cross A., Flesch L., Bedel A., Hickey V. (2020). Improving Oral Health and Modulating the Oral Microbiome to Reduce Bloodstream Infections from Oral Organisms in Pediatric and Young Adult Hematopoietic Stem Cell Transplantation Recipients: A Randomized Controlled Trial. Biol. Blood Marrow Transplant..

[34] Petersen C., Round J.L. (2014). Defining Dysbiosis and Its Influence on Host Immunity and Disease. Cell. Microbiol..

[35] Ryan M.P., Adley C.C. (2014). Ralstonia Spp.: Emerging Global Opportunistic Pathogens. Eur. J. Clin. Microbiol. Infect. Dis..

[36] Oku S., Takeshita T., Futatsuki T., Kageyama S., Asakawa M., Mori Y., Miyamoto T., Hata J., Ninomiya T., Kashiwazaki H. (2020). Disrupted Tongue Microbiota and Detection of Nonindigenous Bacteria on the Day of Allogeneic Hematopoietic Stem Cell Transplantation. PLoS Pathog..

[37] Lang P.M., Jacinto R.C., Pizzol T.S.D., Ferreira M.B., Montagner F. (2016). Resistance Profiles to Antimicrobial Agents in Bacteria Isolated from Acute Endodontic Infections: Systematic Review and Meta-Analysis. Int. J. Antimicrob. Agents.

[38] Liu G., Wu C., Abrams W.R., Li Y. (2020). Structural and Functional Characteristics of the Microbiome in Deep-Dentin Caries. J. Dent. Res..

[39] Celik Z.C., Cakiris A., Abaci N., Yaniikoglu F., Ilgin C., Ekmekci S.S., Celik H., Tagtekin D. (2021). The Complex Microbiome of Caries-Active and Caries-Free Supragingival Plaques in Permanent Dentition. Niger. J. Clin. Pract..

[40] Robertson D., Smith A.J. (2009). The Microbiology of the Acute Dental Abscess. J. Med. Microbiol..

[41] Hiranmayi K.V., Sirisha K., Rao M.V.R., Sudhakar P. (2017). Novel Pathogens in Periodontal Microbiology. J. Pharm. Bioallied Sci..

[42] Gajdács M., Urbán E. (2020). The Pathogenic Role of *Actinomyces* spp. and Related Organisms in Genitourinary Infections: Discoveries in the New, Modern Diagnostic Era. Antibiotics.

[43] Fakhruddin K.S., Ngo H.C., Samaranayake L.P. (2018). Cariogenic Microbiome and Microbiota of the Early Primary Dentition: A Contemporary Overview. Oral Dis..

[44] Bor B., Bedree J.K., Shi W., McLean J.S., He X. (2019). Saccharibacteria (Tm7) in the Human Oral Microbiome. J. Dent. Res..

[45] Jo R., Nishimoto Y., Umezawa K., Yama K., Aita Y., Ichiba Y., Murakami S., Kakizawa Y., Kumagai T., Yamada T. (2019). Comparison of Oral Microbiome Profiles in Stimulated and Unstimulated Saliva, Tongue, and Mouth-Rinsed Water. Sci. Rep..

