# Supplementary material for: Long-Term Analysis of Resilience of the Oral Microbiome in Allogeneic Stem Cell Transplant Recipients
Source: Microorganisms. 2022 Mar 29;10(4):734. doi: 10.3390/microorganisms10040734 (PMC9030553; doi:10.3390/microorganisms10040734)
Supplement: Supplementary file 1 [file microorganisms-10-00734-s001.zip › microorganisms-1601680-supplementary.pdf]

Article

Long-Term Analysis of Resilience of the Oral Microbiome in Allogeneic Stem Cell Transplant Recipients

Alexa M.G.A. Laheij [1,2,3,*], Frederik R. Rozema [1,3], Michael T. Brennan [4], Inger von Bültzingslöwen [5], Stephanie J.M. van Leeuwen [6], Carin Potting [7], Marie-Charlotte D.N.J.M. Huysmans [6], Mette D. Hazenberg [8], Bernd W. Brandt [2], Egija Zaura [2], Mark J. Buijs [2], Johannes J. de Soet [2], Nicole N.M. Blijlevens [7] and Judith E. Raber-Durlacher [1,3]

1 Department of Oral Medicine, Academic Centre for Dentistry Amsterdam, University of Amsterdam and Vrije Universiteit Amsterdam, Amsterdam, The Netherlands; a.laheij@acta.nl; fred.rozema@acta.nl; j.raber.durlacher@acta.nl
2 Department of Preventive Dentistry, Academic Centre for Dentistry Amsterdam, University of Amsterdam and Vrije Universiteit Amsterdam, Amsterdam, The Netherlands; a.laheij@acta.nl; b.brandt@acta.nl; e.zaura@acta.nl; m.buijs@acta.nl; j.d.soet@acta.nl
3 Department of Oral and Maxillofacial Surgery, Amsterdam UMC, University of Amsterdam, Amsterdam, The Netherlands; a.laheij@acta.nl; fred.rozema@acta.nl; j.raber.durlacher@acta.nl
4 Department of Oral Medicine, Atrium Health Carolinas Medical Centre, Charlotte, NC; Department of Otolaryngology/Head and Neck Surgery, Wake Forest University School of Medicine, Winston-Salem, NC, United States of America; Mike.Brennan@atriumhealth.org
5 Department of Oral Microbiology and immunology, Institute of Odontology, The Sahlgrenska Academy, University of Gothenburg, Gothenburg, Sweden; ingervonb@hotmail.com
6 Department of Dentistry, Radboud Institute for Health Sciences, Radboud university medical center, Nijmegen, The Netherlands; Stephanie.vanLeeuwen@radboudumc.nl; Marie-Charlotte.Huysmans@radboudumc.nl
7 Department of Hematology, Radboud Institute for Health Sciences, Radboud university medical center, Nijmegen, The Netherlands; c.potting@hotmail.com; Nicole.Blijlevens@radboudumc.nl
8 Department of Hematology, Amsterdam UMC, University of Amsterdam, Amsterdam, The Netherlands; m.d.hazenberg@amc.uva.nl
* Correspondence: a.laheij@acta.nl

Table S1. Sequencing output control samples.

mean ctrls reads per sample	mean exp reads per sample	ratio control/total control reads	ratio exp/total exp reads	taxonomy
3.72	0.16	0.165	9.52E-06	f__Comamonadaceae
2.69	0.07	0.119	4.21E-06	f__Comamonadaceae; g__Leptothrix; s__sp._oral_taxon_025
1.69	0	0.075	0	c__Alphaproteobacteria
1.45	0.08	0.064	4.39E-06	f__Caulobacteraceae; g__Caulobacter; s__sp._oral_taxon_002

1.41	0.08	0.063	4.58E-06	f__Bradyrhizobiaceae; g__Bosea; s__vestrisii
1.14	0.06	0.050	3.30E-06	f__Burkholderiaceae; g__Ralstonia; s__sp._oral_taxon_406
1.03	0.05	0.046	2.75E-06	c__Alphaproteobacteria
1.03	0.02	0.046	9.15E-07	f__Burkholderiaceae
